# Effect of Polyphenols and Zinc Co-Supplementation on the Development of Neoplasms in Rats with Breast Cancer

**DOI:** 10.3390/foods12020356

**Published:** 2023-01-12

**Authors:** Martyna Jastrzębska, Joanna Giebułtowicz, Andrzej K. Ciechanowicz, Robert Wrzesień, Wojciech Bielecki, Barbara Bobrowska-Korczak

**Affiliations:** 1 Department of Bromatology, Warsaw Medical University, S. Banacha 1 Street, 02-097 Warsaw, Poland; 2 Department of Drug Analysis, Warsaw Medical University, S. Banacha 1 Street, 02-097 Warsaw, Poland; 3 Laboratory of Regenerative Medicine, Medical University of Warsaw, S. Banacha 1b Street, 02-097 Warsaw, Poland; 4 Central Laboratory of Experimental Animals, Warsaw Medical University, S. Banacha 1 Street, 02-097 Warsaw, Poland; 5 Department of Pathology and Veterinary Diagnostics, Institute of Veterinary Medicine, Warsaw University of Live Sciences, Nowoursynowska 159c Street, 02-787 Warsaw, Poland

**Keywords:** zinc, apigenin, epicatechin, naringenin, cancer, N6-methyl-2’-deoxyadenosine, 3-methyladenine

## Abstract

The aim of the study was to evaluate the effect of selected polyphenolic compounds: epicatechin, apigenin, and naringenin, administered separately or in combination with zinc (Zn), on the growth and development of the neoplastic process induced by 7,12-dimethylbenz[a]anthracene (DMBA) in rats. The impact of supplementation with the above-mentioned compounds on the content of modified derivatives: 1-methyladenosine, N6-methyl-2’-deoxyadenosine, O-methylguanosine, 7-methylguanine, 3-methyladenine, 1-methylguanine, 2-amino-6,8-dihydroxypurine, and 8-hydroxy-2’-deoxyguanosine in the urine of rats with mammary cancer was also assessed. Female Sprague-Dawley rats divided into 7 groups were used in the study: animals without supplementation and animals supplemented with apigenin, epicatechin, and naringenin separately or in combination with zinc. To induce mammary cancer, rats were treated with DMBA. Modified derivatives were determined by a validated high-performance liquid chromatography coupled to mass spectrometry method. Based on the obtained results, it can be said that supplementation of the animals with naringenin inhibits the development and progression of the neoplastic process in rats treated with 7,12-dimethylbenzanthracene. Neoplastic tumors were found in only 2 of 8 rats (incidence: 25%) and were considered to be at most grade 1 malignancy. The first palpable tumors in the group of animals receiving naringenin appeared two–three weeks later when compared to other groups. The combination of zinc with flavonoids (apigenin, epicatechin, and naringenin) seems to stimulate the process of carcinogenesis. The level of N6-methyl-2’-deoxyadenosine and 3-methyladenine in the urine of rats was statistically significantly higher in the groups supplemented with apigenin, epicatechin, and naringenin administered in combination with Zn than in the groups receiving only polyphenolic compounds. In conclusion, supplementation of rats with selected flavonoids administered separately or in combination with Zn has an impact on the development of neoplasms and the level of modified nucleosides in the urine of rats with breast cancer. Our results raise the question of whether simultaneous diet supplementation with more than one anti-cancer agent may reduce/stimulate the risk of carcinogenesis.

## 1. Introduction

According to the World Health Organization’s (WHO) report, over the past two decades, the number of new cancer cases has nearly doubled, from 10 million in 2000 to 19.3 million in 2020 [1]. In 2020, breast cancer was the most often diagnosed cancer among women worldwide [1]. Breast cancer was diagnosed in about 2 million women around the world and caused 685,000 deaths [2,3]. While only 5–10% of all cancers are caused by genetic predispositions, 90–95% of tumor pathogenesis can be explained by environmental factors or unhealthy lifestyles, including alcohol consumption, unhealthy eating patterns, and obesity [4,5].

In searching for compounds with anti-cancer effects, much attention is paid to studies on polyphenolic compounds, including apigenin (API), epicatechin (EC), and naringenin (NGE) [6,7,8]. The mechanism of action of the aforementioned compounds is complex and not yet fully known. So far, they have been shown to be capable of: preventing mutagenesis, inhibiting tumor cell proliferation, inducing apoptosis, preventing metastasis, and inducing tumor cell differentiation, among other things. There is evidence of their ability to scavenge free radicals, form complexes with metal ions catalyzing the formation of free radicals, and inhibit certain enzymes involved in oxidative processes [6,7,8,9]. However, polyphenolic compounds can also exhibit pro-oxidant activity [10,11]. Therefore, the direction of their action depends on several environmental factors, i.e., the condition of the body, and the dose and timing of use of these compounds [6,7,8,9,10,11].

Currently, more and more attention is devoted to explaining the role of elements in the process of carcinogenesis. Zinc (Zn) is required by more than 300 enzymes and nearly 2000 transcription factors. Moreover, it is associated with multiple human diseases due to its role in neutralizing free radicals and antioxidant properties, including different types of cancer [12,13]. In the available literature, clinical and epidemiological studies indicate a correlation between a lower risk of cancer and the content of Zn in food intake [14,15,16,17,18,19]. These properties of this element are mainly related to antioxidant activity. Furthermore, it should be borne in mind that studies have shown that Zn can influence the immune system, cell differentiation and proliferation, transcription factors, DNA and RNA synthesis and repair, enzyme regulation, intercellular signaling, and stabilization of cell structure and membranes, which are also important during tumorigenesis [14,15,16,17]. Many patients with cancer, especially of the lungs, breast, head, and neck, have a decreased level of Zn in their blood [18,19]. More than two billion patients suffer from Zn deficiency, and people with chronic Zn deficiency have an increased risk of cancer. The problem is the dietary intake of Zn, which is about 10% of the recommended intake [20,21,22,23]. The recommended daily dose of zinc is 8 milligrams (mg) for women and 11 mg for adult men. The best sources of zinc are oysters, red meat, and poultry. Other good sources of Zn are beans, nuts, crab, lobster, whole grains, breakfast cereals, and dairy products. Supplementation and an optimal intake of Zn restore the normal immune response and reduce the risk of cancer. However, the optimal immunostimulatory dose of Zn has not been determined [24]. Despite this, it is still unknown whether the use of two compounds with anticancer activity will enhance the protective effect against this process. Thus, there is a need to study the effect of flavonoids on the cancer process in combination with some chemical elements or metal complexes, such as zinc.

Key processes responsible for epigenetic regulation include DNA methylation, histone modification, and post-transcriptional regulation of genes by non-coding genes. These mechanisms are critical elements in normal cell growth and development, and their modifications contribute to cancer phenotypes, among other things. During carcinogenesis, RNA turnover is accelerated, resulting, among other things, in the formation of modified nucleosides, which can affect changes in the association/dissociation of specific proteins. This mechanism disrupts homeostasis. In urine, abnormal methylated nucleoside concentrations may be associated with higher methyltransferase activity and increased RNA turnover [25]. Modified nucleosides, regarded as indicators for the whole-body turnover of RNAs, are excreted in abnormal amounts in the urine of patients with malignancies. Elevated levels of modified nucleosides have been found in the urine of patients with leukemia and lymphoma, cancer of the lung, esophagus, breast, renal cell carcinoma, ovarian cancer, liver cancer, tumors of the bladder, and colon cancer [25,26,27,28,29,30]. There is evidence that epigenetic modifications, can be influenced by bioactive components of the diet [31,32,33,34,35]. Some studies indicate that, among others, folic acid, selenium, zinc, and vitamin B supplementation have an impact on the concentration of methylated nucleosides [31,32,33,34,35]. However, there is still a lack of data regarding the influence of polyphenolic compounds, including API, EC, and NGE on epigenetic modifications, especially observed under the conditions of neoplastic process development. Further research on the usefulness of modified nucleosides in urine as tumor markers is needed. 

The aim of the study was to evaluate the effect of selected polyphenolic compounds: apigenin, epicatechin, and naringenin, administered separately or in combination with Zn, on the growth and development of the neoplastic process induced by 7,12-dimethylbenz[a]anthracene (DMBA) in rats. The impact of supplementation with the above-mentioned compounds on the content of modified derivatives: 1-methyladenosine, N6-methyl-2’-deoxyadenosine, O-methylguanosine, 7-methylguanine, 3-methyladenine, 1-methylguanine, 2-amino-6,8-dihydroxypurine, and 8-hydroxy-2’-deoxyguanosine in the urine of rats with mammary cancer was also assessed. Understanding the anti-cancer mechanisms of dietary compounds may contribute to a broader view of their role in the prevention of neoplastic diseases and support pharmacological treatment, as well as provide the opportunity to assess the safety of their use.

## 2. Materials and Methods

### 2.1. Laboratory Animals

Female Sprague-Dawley rats (n = 52) were obtained from the Animal Laboratory, Department of General and Experimental Pathology, Medical University of Warsaw. The study was approved by the 2nd Ethical Committee for Animal Experiments in Warsaw (Approval No. WAW2/090/2021 of 2 June 2021). The rats were kept under the standard conditions, i.e., a 12 h light-dark cycle, constant temperature of 22 °C, and free access to food (standard diet: Labofeed H, Żurawia 19, 89–240 Kcynia, Poland) and water. In order to induce the neoplastic process, the animals were treated with 7,12-dimethylbenz[a]anthracene administered with an intragastric tube at a dose of 80 mg/kg of body weight, in rapeseed oil (0.4 mL) at 50 days of rats’ age.

The animals were divided into 7 groups depending on the supplementation used:

- group 1—animals without supplementation (n = 8),
- group 2—animals receiving apigenin (0.35 mg/mL) (aqueous solution) (n = 7),
- group 3—animals receiving apigenin (0.35 mg/mL) in combination with zinc (6.9 mg/mL) (aqueous solution) (n = 7),
- group 4—animals receiving epicatechin (3.5 mg/mL) (aqueous solution) (n = 7),
- group 5—animals receiving epicatechin (3.5 mg/mL) in combination with zinc (6.9 mg/mL) (aqueous solution) (n = 8),
- group 6—animals receiving naringenin (3.5 mg/mL) (aqueous solution) (n = 8),
- group 7—animals receiving naringenin (3.5 mg/mL) and zinc (6.9 mg/mL) (aqueous solution) (n = 7).

**Figure 1:**
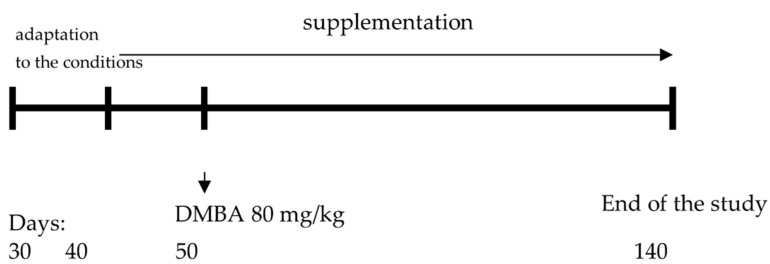
The scheme of the experiment design.

The animals were supplemented with the above-mentioned polyphenolic compounds from the 40th day of life to the 20th week of life (end of the study). The above-mentioned supplements were administered to the animals with an intragastric tube at a volume of 0.4 mL. The doses were established based on the levels of polyphenolic compounds and zinc occurring in supplements available to humans in pharmacies (per animal body weight). In order to maintain the experimental conditions, the animals in group 1 received 0.4 mL of water via an intragastric tube. The scheme of the experiment design is presented in Figure 1.

The animals were examined by palpation during the study to characterize the time course of tumor development. In order to obtain urine samples, each animal was individually placed in a metabolic cage for 24 h. Urine samples were collected once at the end of the study. The samples were stored at a temperature of −70 °C until further analysis.

### 2.2. Histopathological Examination

At day 140, the rats were subjected to decapitation. The resulting tumors were assessed histopathologically. The excised tumors were treated with buffered formalin solution, the next step was dehydration and paraffin sealing. Tumors prepared in this way were cut into pieces 4 µm thick. Tissue sections were stained with hematoxylin and eosin. Evaluation was performed using an Olympus BX43 research microscope (Olympus Europa SE & Co., Hamburg, Germany). Mitoses were counted in slides from randomly selected tumors in 15 fields of view at 40× objective magnification.

### 2.3. Chromatography

The analytical method used is based on high-performance liquid chromatography coupled with mass spectrometry (LC-MS/MS) and was validated in accordance with relevant guidelines [36]. The analytes were determined using multiple reaction monitoring modes on an Agilent 1260 Infinity (Agilent Technologies, Santa Clara, CA, USA) instrument coupled to a QTRAP 4000 (AB Sciex, Framingham, MA, USA). MRM transitions, declustering potential (DP), and collision energy (CE) were as follows: O-methylguanosine: 298 > 152 (DP = 51 V, CE = 17 V); 1-methyladenosine: 282 > 55 (DP = 66 V, CE = 87 V); 7-methylguanine: 166 > 79 (DP = 96 V, CE = 43 V); 3-methyladenine: 150 > 123 (DP = 86 V, CE = 31 V); 1-methylguanine: 166 > 135 (DP = 81 V, CE = 31 V); N6-methyl-2-deoxyadenosine: 266 > 150 (DP = 61 V, CE = 23 V), 8-hydroxy-2′-deoxyguanosine; 284 > 168 (DP = 46 V, CE = 19 V); and 2-amino-6,8-dihydroxypurine 168 > 140 (DP = 81 V, CE = 23 V). Chromatographic separation was achieved using a SeQuant^®^ ZIC^®^-HILIC (50 × 2.1 mm, 5 µm, Merck) column, at 25 °C with a flow rate of 0.5 mL min^−1^. The mobile phases were 20 mM ammonium acetate (eluent A) and acetonitrile with 0.2% formic acid (eluent B). The gradient (%B) was as follows: 0 min 95%; 1 min 95%; 7 min 50%; 8 min 50%. The injection volume was 5 µL. Reference standards, i.e., 1-methyladenine, 3-methyladenine, 7-methylguanine, 1-methylguanine, 1-methyladenosine, 7-methylguanosine, O-methyl-guanosine, N6-methyl-2′-deoxyguanosine, 8-hydroxy-2′-deoxyguanosine, 2-amino-6,8-dihydroxypurine, as well as an internal standard (tubercidin), were purchased from Sigma-Aldrich (St Louis, MO, USA). Urine samples (0.1 mL) were mixed with tubercidin (0.1 mL, 1 µg/mL) and acetonitrile (0.6 mL), vortexed at high speed (3 min) and centrifuged (5 min at 10,000× *g*) prior to injection into the chromatograph.

### 2.4. Determination of Creatinine Content

The level of the modified nucleosides and bases in urine was standardized by conversion to the creatinine level. The latter was determined in urine samples with a commercial creatinine test (“CREATININE Liquicolor Jaffe-Reaction Photometric Colorimetric Test for Kinetic Measurements. Method without Deproteinisation” by Human Gesellschaft für Biochemica und Diagnostica mbH, Max-Planck-Ring 21 65,205 Wiesbaden Germany) based on Jaffe’s reaction.

### 2.5. Statistics

Statistical analysis was performed using the GraphPad Prism 9 statistical software. (https://www.graphpad.com/scientific-software/prism/, accessed on 18 November 2022). The results of the study were compared using analysis of variance (ANOVA). In order to verify the results of tumor incidence in the DMBA-treated rats, the relative risk (RR) calculation was used. The test probability at the level of *p* < 0.05 was considered significant.

## 3. Results

**Figure 2:**
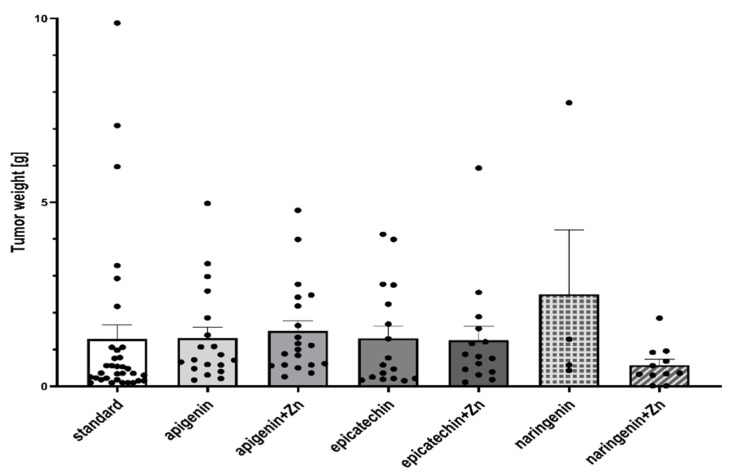
Tumor weight (g) of rats treated with 7,12-dimethylbenz[a]anthracene in relation to supplementation. There is no significant difference between groups.

**Figure 3:**
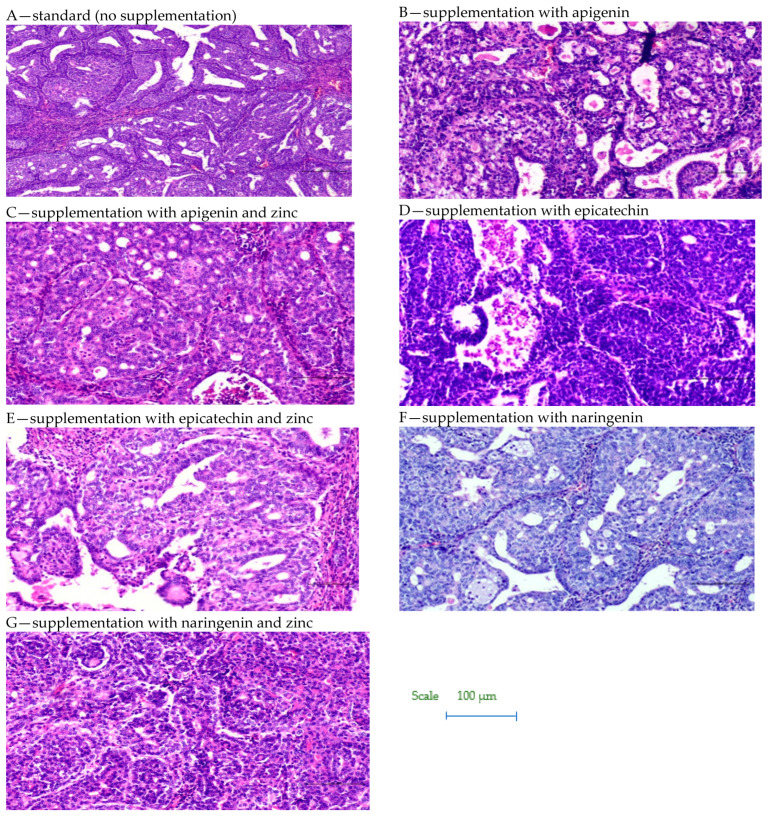
Biopsy images of tumor samples with 10× magnification.

Based on the study, it was shown that supplementation of animals with selected dietary components influences the intensity of the tumor process in rats (Table 1, Figure 2 and Figure 3). It was shown that supplementing animals with NGE statistically significantly inhibited the development of the neoplastic process: the incidence was 25%, the number of tumors was 0–2, and they were characterized by a 0–1 degree of malignancy. In the case of animals supplemented with NGE in combination with Zn, the tumor incidence was 100%. Moreover, the first palpable tumors in this group were observed at week 17 (as in the case of NGE), the average weight of tumors was significantly lower (in the range of 0.01–1.85), and the number of tumors per rat was 1–2. Tumors obtained from animals supplemented with NGE and NGE in combination with Zn showed vesicular–vesicular structures, similar to a differentiated neoplasm with very low malignancy (*adenocarinoma* 0–1 grade of malignancy). In animals without supplementation, the incidence was 100%, and the first palpable tumors were found at week 15. The observed number of tumors per rat ranged from 2–8, and their weight ranged from 0.10–9.89 g. Tumors in the animals receiving a diet without supplementation were classified as adenocarcinoma (*adencarcinoma* I degree), the cells showed a tendency to proliferate, and massive proliferation was observed. In our previous research, it was found, that in the case of rats supplemented with zinc, the incidence was 100%, the number of tumors per rat ranged from 1–5, and those tumors were classified as I degree *adencarcinoma* [37,38].

However, in the case of API- and EC-supplemented animals given in combination with Zn, the first palpable tumors were found a week earlier (week 14), than in the case of animals without supplementation and those supplemented with API and Zn, and 3 weeks earlier with respect to the other study groups. In the group of API-Zn-supplemented animals, grade 2–3 malignant tumors were found (grade 1–2 in the other groups). Tumor cells obtained from API-Zn-supplemented animals showed high differentiation, with numerous mitoses. In the case of animals supplemented with API in combination with Zn, there was a statistically significant increase in tumor cell proliferation (number of mitoses: 1.91 ± 0.13), compared to animals supplemented with API separately (1.04 ± 0.07).

**Figure 4:**
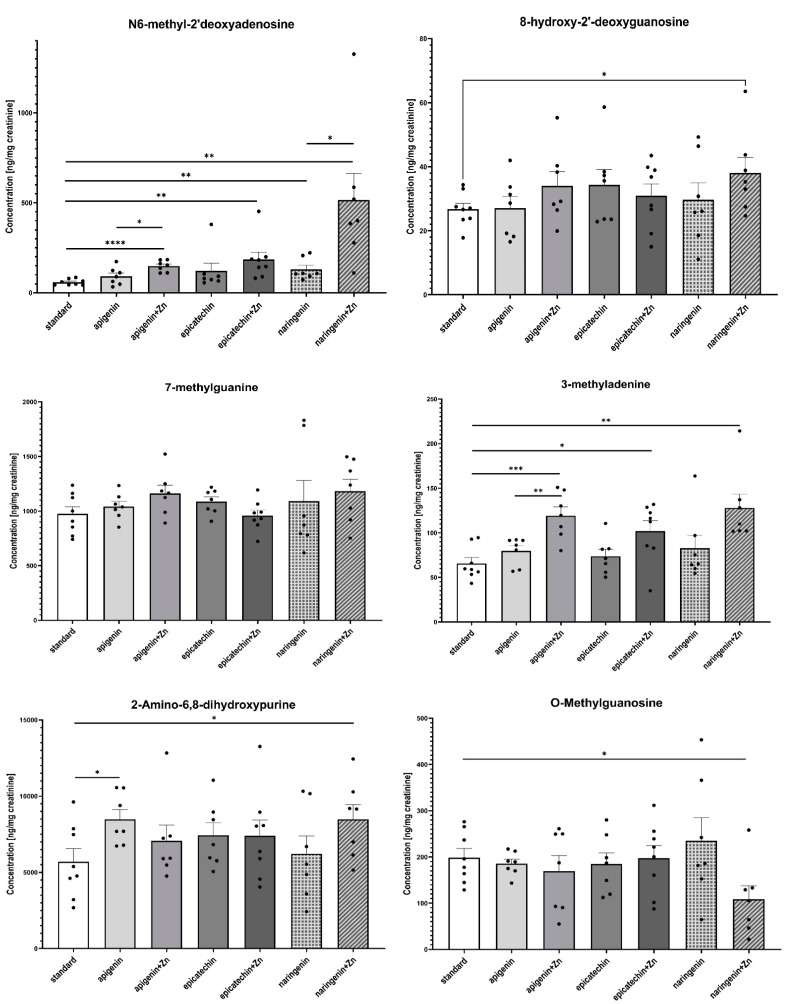

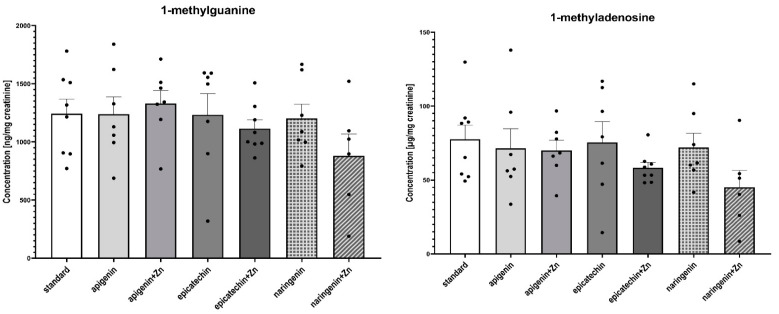
The content of selected methyl derivatives in urine of rats treated with 7,12-dimethylbenz[a]anthracene in relation to supplementation. The differences were considered significant at: *—*p* < 0.05; **—*p* < 0.01; ***—*p* < 0.001; ****—*p* < 0.0001.

**Figure 5:**
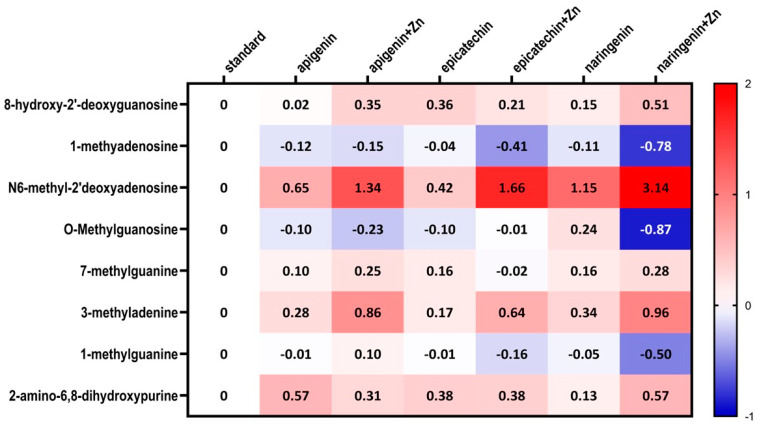
Heatmap of changes of the selected methyl nucleosides derivatives contents depending on the type of supplementation used compared to standard group (i.e., group 1).

The ANOVA test analysis showed that there is a statistically significant influence of the supplementation on the content of N6-methyl-2’-deoxyadenine (*p* = 0.0001) and 3-methyladenine (*p* = 0.0006) in the urine of rats under neoplastic conditions. It was shown that animals supplemented with flavonoids in combination with Zn were characterized by a significantly higher content of N6-methyl-2’-deoxyadenosine and 3-methyladenine in urine, compared to animals on a standard diet without supplementation as well as those supplemented with flavonoids (apigenin, epicatechin, or naringenin separately) (Figure 4, Table 2). Figure 5 shows the observed average results for each group compared to group 1 (without supplementation).

## 4. Discussion

Regarding the study results, the significant inhibitory effect of naringenin on the development of DMBA-induced neoplastic processes in rats deserves special attention. Neoplastic tumors were found in only 2 out of 8 rats (incidence: 25%) and were considered to be grade 0–1 malignancy. The first palpable tumors in the group of animals receiving NGE were observed two weeks later (week 17) in comparison to both, the group receiving a standard diet and the group receiving API. Moreover, three weeks later than in the group of animals receiving API in combination with Zn and EC in combination with Zn. Similar study results were obtained by other authors [39,40,41,42,43,44,45,46,47]. NGE showed its positive effect on the neoplastic process in studies for: colon cancer [39], breast cancer [40], lung cancer [41], cervical cancer [42], bladder cancer [43], prostate cancer [44], gastric cancer [45], pancreatic cancer [46], and epidermoid carcinoma [47]. NGE is able to decrease cancer cell proliferation as well as induce cell cycle arrest and apoptosis in a variety of cancer cell lines. Thus, it has potential anticancer activity [48]. Moreover, NGE is involved in the mechanisms of inhibiting both inflammation and survival signaling pathways, such as NF-κB, MAPK, or AKT [35]. Moreover, it has been shown that NGE reduced adiposity and ameliorated adipose tissue inflammation with a moderate inhibitory effect on tumor growth in obese ovariectomized mice [49]. Another study showed that NGE reduces lung metastasis in a breast cancer resection model. It was administered orally and significantly decreased the number of metastatic tumor cells in the lung and extended the life span of tumor-resected mice. NGE inhibits the outgrowth of metastases after surgery via regulating host immunity. Thus, NGE can be an ideal surgical adjuvant therapy for breast cancer patients [50].

The findings of the study revealed that supplementation of the animals with polyphenolic compounds in combination with Zn caused an increase in the development of the neoplastic process compared to the animals receiving API, EC, and NGE. The first palpable tumors in the animals receiving API in combination with Zn were observed at week 14, which means a week earlier than in the animals receiving only API and standard diet. The tumors were graded as II and III histopathological malignancies, and the weight of the tumors ranged from 0.36 to 4.78 g (average: 1.50 ± 1.24 g). On the contrary, the animals on a standard diet and the ones supplemented with API separately were characterized by grade I tumor malignancy and tumor weights of 0.10–9.89 g (average: 1.28 ± 2.24 g); 0.21–3.33 g (mean: 1.31 ± 1.29 g), respectively. In the case of rats supplemented with zinc, the incidence was 100%, and the number of tumors per rat ranged from 1–5 tumors, which were classified as I-degree *adenocarcinoma* [37,38]. Interestingly, supplementation of the animals with EC in combination with Zn also stimulated the initiation of the tumorigenic process—the first palpable tumors in this group were observed 3 weeks earlier (week 14) than in the group of animals supplemented with EC separately, and a week earlier than in the case of the animals without supplementation or supplemented with zinc [38,39]. However, the first tumors in the group of animals supplemented with EC in combination with Zn were observed relatively early (in the 14th week), and they were graded as I degree. Their weight ranged from 0 to 5.93 g (average: 1.26 ± 1.46 g), and the incidence in this group of animals was 75% (6 out of 8 rats). In the case of animals supplemented with epicatechin separately, the first palpable tumors in the group were observed at week 17, and the incidence was 100%. Those tumors were graded as II-degree malignancy, and their weight ranged from 0.19–3.99 (average: 1.31 ± 1.37). Relating these results to the scientific research background, we see examples of studies in which the addition of trace elements to other anticancer agents was responsible for the stimulation of tumorigenesis. Bobrowska-Korczak et al. [38] found that the application of combined diet supplementation with zinc ions and resveratrol considerably promoted the rate of carcinogenesis and increased the number of DMBA-induced mammary tumors. Gontero et al. [51] showed that co-supplementation of lycopene, green tea catechins (GTCs), and selenium in men with multifocal high-grade prostatic intraepithelial neoplasia (mHGPIN) and/or atypical small acinar proliferation (ASAP) was associated with a higher incidence of prostate cancer (PCa) at re-biopsy and expression of microRNAs implicated in PCa progression at molecular analysis. According to their results, dietary supplementation at the highest non-toxic doses of selenium, lycopene, and GTCs exerted a negative rather than positive impact on HGPIN/ASAP patients in terms of the risk of PCa development. The report of the Selenium and Vitamin E Cancer Prevention Trial (SELECT) found no reduction in the risk of prostate cancer with either selenium or vitamin E supplements [52]. Studies have shown that selenium can interfere with the proper functioning of zinc. The two elements interact, which can lead to a loss of the antioxidant properties of Zn. Selenium affects the release of Zn and may alternately have a prooxidant or antioxidant function [53]. Disturbed zinc homeostasis disrupts the normal oxidoreductive metabolism of cells by affecting the metallothionein system. The consequence of these processes is dysregulation of the melatonin system. A further effect of dysregulation of the metallothionein system is the modulation of transcription of the DNA repair gene p53. DNA repair, cell cycle arrest, and apoptosis are inactivated. Quesada and colleagues [54] have found that epigallocatechin gallate (EGCG) as well as grape-seed procyanidin extract (GSPE) enhance the expression of Zn transporters ZIP1 and ZIP4 and inhibit the expression of Zn-binding metallothioneins and Zn exporter ZnT1 in the hepatocarcinoma cell line HepG2. Results mostly focus on the separate effects of these two elements on different cell types, tissues, and organs, but their combined effects are largely unknown. However, Malhotra [55] has shown that combined supplementation of curcumin and resveratrol increases the intra-tumoral Zn level and controls inflammation by COX-2 and cell cycle arrest by p21 during lung carcinogenesis in mice. It is possible that natural compounds may have abilities to influence Zn homeostasis; however, the usefulness of supplementation of the above-mentioned compounds in combination with Zn for cancer management still remains unclear. The effect of Zn and polyphenol co-supplementation in the context of the treatment of neoplastic diseases probably depends on the use of a specific dose, the physiological state of the tested organism, and the diet. The mechanism and effect of their action at the stage of initiation and progression of the neoplastic process are still not fully known.

The second part of the study was to evaluate the effect of supplementation with selected polyphenolic compounds: epicatechin, apigenin, and naringenin, administered separately or in combination with zinc, on the content of: 3-methyladenine, 7-methylguanine, 1-methylguanine, 1-methyladenosine, 7-methylguanosine, O-methylguanosine, N6-methyl-2’-deoxyguanosine in the urine of rats under neoplastic conditions. Nucleosides, nucleobases, and their modified analogs have drawn the interest of scientists for years. Urinary excretion of the methylated compounds is an indicator of the whole-body turnover and/or degradation of the methylated RNA, especially transfer ribonucleic acid (tRNA) [25,56]. tRNA is a type of RNA that regulates protein synthesis. Due to the fact that 80–90% of the dry mass of cells is represented by proteins, the level of translation is a determinant of cell growth and differentiation. In addition, an uncharged tRNA acts as a signaling molecule in the regulation of numerous metabolic and cellular processes. Moreover, a tRNA functions as an effective scavenger of cytochrome *c*, which is consistent with its role in the regulation of apoptosis [57]. All these functions of tRNA have led to the proposal of its role in cancer development. Certainly, it is noteworthy, that we still need more research to assess the value of modified nucleosides as tumor markers. The above-mentioned compounds are considered promising bioindicators for tracking the development of the cancer process, including early detection and cancer prevention. Some studies have shown that the modified RNA molecules are metabolized, but not reincorporated into tRNA. Instead, tRNA is excreted in the urine, which reflects the process of RNA degradation in an organism [25,56]. In our studies, it was found that the rats additionally supplemented with Zn showed a statistically significantly higher content of N6-methyl-2’-deoxyadenosine (m^6^dA) and 3-methyladenine (3-MA) in the urine, compared to animals receiving a standard diet as well as those supplemented with API, EPI, or NGE separately. It is particularly interesting because, in the case of our study, supplementation of animals with two anti-cancer agents seems to stimulate the neoplastic process instead of enhancing inhibition, which was anticipated for the experiment. Zn given as an additional agent to polyphenolic compounds seems to stimulate the development of the neoplastic process. 

The bulk of genomic m^6^dA originates from ribo-*N*^6^-methyladenosine (N^6^-methyladenosine) which is processed via the nucleotide-salvage pathway and misincorporated by DNA polymerases [58,59,60,61]. 6mA has a major impact on RNA metabolism through nuclear export, decay, alternative splicing, and translation. 6mA is an important factor in the proper functioning of the body and is involved in many processes such as sperm formation (spermatogenesis), thermoregulation, T-cell homeostasis, proliferation, and stem cell differentiation. Balance of the 6mA level is regulated by 6mA demethylases, 6mA methyltransferases (METTL), and 6mA-binding protein. There are numerous studies highlighting the role of 6mA in regulating gene expression and the stress response, chromatin organization, and tumorigenesis [61,62,63,64]. Recently, more and more studies have shown that 6mA methylation has a profound effect on tumorigenesis and progression [65]. METTL3 was found to be upregulated in breast cancer tissues and cells, where it promoted 6mA modification of the 3′ UTR of B-cell/lymphoma 2 (BCL-2) mRNA. This modification resulted in the upregulation of BCL-2 expression, thereby promoting cell proliferation, inhibiting apoptosis, and promoting tumor growth [66,67]. Upregulation of 6mA methylation in breast cancer has been noted, but the mechanism of this action is still unclear. The results show that the concentration of 2′-O-methyladenosine (A_m_), *N*^6^-methyladenosine (6mA), *N*^6^,2′-O-dimethyladenosine (m^6^A_m_) was increased, whereas the concentration of *N*^1^-methyladenosine (m^1^A) was decreased in the urine of breast cancer patients compared with the healthy controls [68,69]. 

Next, the nucleoside derivative important for this study is 3-methyladenine (3-MA; 6-amino-3-methylpurine). It is described in numerous studies as an autophagy inhibitor [70]. 3-MA inhibits autophagy by blocking autophagosome formation via the inhibition of type III phosphatidylinositol 3-kinases (PI-3K). Although the role of autophagy in properly functioning cells is well understood, its role in cancer cells is still a subject of research. There is no direct connection between autophagy and tumorigenesis. The inhibition of autophagy supports the process of neoplastic transformation in many types of cancer and is responsible for their progression. Nevertheless, the autophagy process is a mechanism that cancer cells need to survive under conditions of oxidative stress and restricted nutrient supply. Thus, autophagy plays a dual role in the cancer process, i.e., it can inhibit tumor progression but also stimulate it by allowing tumor cells to survive in unfavorable environmental conditions. In addition to its role in autophagy, 3-MA has been implicated in cancer therapy. It has been revealed that 3-MA suppresses the invasion of highly metastatic cancer cells through the inhibition of class I and II PI3K. Alkylating agents, such as 3-MA, strongly block replication [71,72,73]. Further studies demonstrated that 3-MA can induce caspase-dependent cell death, which is independent of autophagy inhibition [74].

## 5. Conclusions

In conclusion, supplementation with NGE has been shown to inhibit the development and progression of the neoplastic process in rats treated with 7,12-dimethylbenzantharcene. Based on the obtained results, it can be said that the combination of flavonoids with Zn seems to stimulate the process of carcinogenesis and has an impact on the content of methylated nucleosides in the urine of rats. The level of N6-methyl-2’-deoxyadenosine and 3-methyladenine was statistically significantly higher in the groups supplemented with API, EC, and NGE administered in combination with Zn than in the groups receiving only polyphenolic compounds. Further studies are needed in this field. The presented results call into question if diet supplementation with more than one anti-cancer agent at the same time is likely to reduce the expected results of inhibiting the risk of carcinogenesis.

## Figures and Tables

**Table 1:** Mammary tumor formation in individual experimental groups with 7,12-dimethylbenz[a]anthracene (DMBA) in relation to supplementation.

Experimental Group	Week	Tumor Incidence (%)	Numer of Tumors in One Rat	Tumor Weight Mean + SD (Range) (g)	Tumor Grade	Number of Mitoses #
Standard (no supplementation)	15	100% (8/8) ^a^	2–8	1.28 ± 2.24 (0.10–9.89)	1 grade	1.84 ± 0.72 ^ac^
Supplementation with apigenin	15	100% (7/7) ^a^	1–4	1.31 ± 1.29 (0.21–3.33)	1 grade	1.04 ± 0.07 ^b^
Supplementation with apigenin and zinc	14	100% (7/7) ^a^	1–4	1.50 ± 1.24 (0.36–4.78)	2–3 grade	1.91 ± 0.13 ^a^
Supplementation with epicatechin	17	100% (7/7) ^a^	1–4	1.31 ± 1.37 (0.19–3.99)	2 grade	1.67 ± 0.1 ^a^
Supplementation with epicatechin and zinc	14	75% (6/8) ^a^	0–5	1.26 ± 1.46 (0–5.93)	1 grade	1.69 ± 0.11 ^a^
Supplementation with naringenin	17	25% (2/8) ^b^	0–2	2.50 ± 3.49 (0.43–7.71)	0–1 grade	1.4 ± 0.67 ^abc^
Supplementation with naringenin and zinc	17	100% (7/7) ^a^	1–2	0.58 ± 0.53 (0.01–1.85)	0–1 grade	1.40 ± 0.27 ^c^

#—Mitoses were counted in slides from randomly selected tumors in 15 fields of view with a 40X magnification objective. Data are expressed as mean ± SD. ^a,b,c^—values with different superscripts in rows. All differences were considered significant at *p* < 0.05.

**Table 2:** The results of significance differences among means of experimental groups.

Marker	Apigenin + Zn vs. Apigenin vs. Standard	Epicatechin + Zn vs. Epicatechin vs. Standard	Naringenin + Zn vs. Naringenin vs. Standard
N6-methyl-2’deoxyadenosine	0.0003	0.0458	0.0019
8-hydroxy-2’-deoxyguanosine	0.2566	0.3464	0.1584
7-methylguanine	0.1409	0.2334	0.5128
3-methyladenine	0.0002	0.0215	0.0058
2-amino-6,8-dihydroxypurine	0.0915	0.3234	0.1361
O-methylguanosine	0.6631	0.9080	0.0519
1-methylguanine	0.8545	0.7410	0.2002
1-methyladenosine	0.8573	0.3131	0.0931

The differences were considered significant if *p* < 0.05.

## Data Availability

The data used to support the findings of this study can be made available by the corresponding author upon request.

## References

[1] World Health Organization (2021). Breast Cancer Now Most Common Form of Cancer: WHO Taking Action. https://www.who.int/news/item/03-02-2021-breast-cancernow-most-com-mon-form-of-cancer-who-taking-action.

[2] World Health Organization (2021). Breast Cancer. https://www.who.int/news-room/fact-sheets/detail/breast-cancer.

[3] Cancer Today (2020). International Agency for Research on Cancer (IARC). https://gco.iarc.fr/today/online-analysis.

[4] Reddy L., Odhav B., Bhoola K.D. (2003). Natural products for cancer prevention: A global perspective. Pharmacol. Therapeut.

[5] Seiler A., Chen M.A., Brown R.L., Fagundes C.P. (2018). Obesity, Dietary Factors, Nutrition, and Breast Cancer Risk. Curr. Breast Cancer Rep..

[6] Yan C., Yang Q., Shen H.M., Spitsbergen J.M., Gong Z. (2017). Chronically high level of tgfb1a induction causes both hepatocellular carcinoma and cholangiocarcinoma via a dominant Erk pathway in zebrafish. Oncotarget.

[7] Javed Z., Sadia H., Iqbal M.J., Shamas S., Malik K., Ahmed R., Raza S., Butnariu M., Mar-tins N., Rad J. (2021). Apigenin role as cell-signaling pathways modulator: Implications in cancer pre-vention and treatment. Cancer Cell Int..

[8] Bernatoniene J., Kopustinskiene D.M. (2018). The Role of Catechins in Cellular Responses to Oxi-dative Stress. Molecules.

[9] Slika H., Mansour H., Wehbe N., Nasser S.A., Iratni R., Nasrallah G., Shaito A., Ghadar T., Kobeissy F., Eid A.H. (2022). Therapeutic potential of flavonoids in cancer: ROS-mediated mecha-nisms. Biomed. Pharmacother..

[10] Akyuz E., Başkan K.S., Tutem E., Apak R. (2017). Novel protein-based solid-biosensor for deter-mining pro-oxidant activity of phenolic compounds. J. Agric. Food Chem..

[11] Castañeda-Arriaga R., Pérez-González A., Reina M., Alvarez-Idaboy J.R., Galano A. (2018). Comprehensive investigation of the antioxidant and pro-oxidant effects of phenolic compounds: A double-edged sword in the context of oxidative stress?. J. Phys. Chem. B.

[12] Jarosz M., Olbert M., Wyszogrodzka G., Młyniec K., Librowski T. (2017). Antioxidant and anti-inflammatory effects of zinc. Zinc-de-Pendent NF-κB Signal. Inflammopharmacol..

[13] Fukada T. (2015). Zinc biology and zinc signaling. Biomed. Res. Trace. Elem..

[14] Stepien M., Hughes D.J., Hybsier S., Bamia C., Tjønneland A., Overvad K., Aret A., His M., Boutron-Ruault M.-C., Katzke V. (2017). Circulating copper and zinc levels and risk of hepatobiliary cancers in Europeans. Br. J. Cancer.

[15] Chasapis C.T., Loutsidou A.C., Spiliopoulou C.A., Stefanidou M.E. (2012). Zinc and human health: An update. Arch. Toxicol..

[16] Fraker P.J., King L.E., Laakko T., Vollmer T.L. (2000). The dynamic link between the integrity of the immune system and zinc status. J. Nutr..

[17] Liaw K.Y., Lee P.H., Wu F.C., Tsai J.S., Lin-Shiau S.Y. (1997). Zinc, copper, and superoxide dis-mutase in hepatocellular carcinoma. Am. J. Gastroenterol..

[18] Kumar R., Razab S., Prabhu K., Ray S., Prakash B. (2017). Serum butyrylcholinesterase and zinc in breast cancer. J. Cancer Res. Ther..

[19] Okunade K.S., Dawodu O.O., Salako O., Osanyin G.E., Okunowo A.A., Anorlu R.I. (2018). Com-parative analysis of serum trace element levels in women with invasive cervical cancer in Lagos, Nigeria. Pan Afr. Med. J..

[20] Wessells K.R., Singh G.M., Brown K.H. (2012). Estimating the Global Prevalence of Inadequate Zinc Intake from National Food Balance Sheets: Effects of Methodological Assumptions. PLoS ONE.

[21] Brown K.H., Hambidge K.M., Ranum P. (2010). Zinc Fortification Working Group Zinc fortification of cereal flours: Current recommendations and research needs. Food Nutr. Bull..

[22] Prasad A.S. (2008). Zinc in human health: Effect of zinc on immune cells. Mol. Med..

[23] Prasad A.S. (2014). Impact of the discovery of human zinc deficiency on health. J. Trace. Elem. Med. Biol..

[24] Dhawan D.K., Chadha V.D. (2010). Zinc: A promising agent in dietary chemoprevention of cancer. Indian J. Med. Res..

[25] Seidel A., Brunner S., Seidel P., Fritz G.I., Herbarth O. (2006). Modified nucleosides: An accurate tumour marker for clinical diagnosis of cancer, early detection and therapy control. Br. J. Cancer.

[26] Sasco A.J., Rey F., Reynaud C., Bobin J.Y., Clavel M., Nivelau A. (1996). Breast cancer prognostic significance of some modified urinary nucleosides. Cancer Lett..

[27] Zheng Y.F., Kong H.W., Xiong J.H., Lv S., Xu G.W. (2005). Clinical significance and prognostic value of urinary nucleosides in breast cancer patients. Clin. Biochem..

[28] McEntire J.E., Kuo K.C., Smith M.E., Stalling D.L., Richens J.W., Zumwalt R.W., Gehrke C.W., Papermaster B.W. (1989). Classification of lung cancer patients and controls by chromatography of modified nucleosides in serum. Cancer Res..

[29] Oerlemans F., Lange F. (1986). Major and modified nucleosides as markers in ovarian cancer: A pilot study. Gynecol. Obstet. Investig..

[30] Holstege A., Pauw M., Häring T., Kirchner R., Pausch J., Gerok W. (1986). Die Wertigkeit einer erhöhten Urinausscheidung modifizierter Nukleoside als Tumormarker beim Kolonkarzinom. Verh. Dtsch. Ges. Inn. Med..

[31] Speckmann B., Schulz S., Hesse D., Schumacher F., Kleuser B., Geisel J., Obeid R., Grune T., Kipp A.P. (2017). Selenium increases hepatic DNA methylation and modulates one-carbon metabolism in the liver of mice. J. Nutr. Biochem..

[32] Uthus E.O., Ross S.A., Davis C.D. (2006). Differential effects of dietary selenium (Se) and folate on methyl metabolism in liver and colon of rats. Biol. Trace Elem. Res..

[33] Wolffe A.P., Matzke M.A. (1999). Epigenetics: Regulation through repression. Science.

[34] Jaenisch R., Bird A. (2003). Epigenetic regulation of gene expression: How the genome integrates intrinsic and environmental signals. Nat. Genet..

[35] Mathers J.C. (2006). Nutritional modulation of ageing: Genomic and epigenetic approaches. Mech. Ageing Dev..

[36] Raćkowska E., Bobrowska-Korczak B., Giebułtowicz J. (2019). Development and validation of a rapid LC-MS/MS method for determination of methylated nucleosides and nucleobases in urine. J. Chromatogr. B.

[37] Bobrowska-Korczak B., Skrajnowska D., Tokarz A. (2013). Effect of zinc and copper supplementation on the prognostic value of urinary 5-methyl-2’-deoxycytidine in DMBA-induced carcinogenesis in rats. Cancer Biomark..

[38] Bobrowska-Korczak B., Skrajnowska D., Tokarz A. (2012). The effect of dietary zinc-and poly-phenols intake on DMBA-induced mammary tumorigenesis in rats. J. Biomed. Sci..

[39] Frydoonfar H.R., McGrath D.R., Spigelman A.D. (2003). The variable effect on proliferation of a colon cancer cell line by the citrus fruit flavonoid Naringenin. Colorectal. Dis..

[40] Zhang F., Dong W., Zeng W., Zhang L., Zhang C., Qiu Y., Wang L., Yin X., Zhang C., Liang W. (2016). Naringenin prevents TGF-β1 secretion from breast cancer and suppresses pulmonary metastasis by inhibiting PKC activation. Breast Cancer Res..

[41] Wadhwa R., Paudel K.R., Chin L.H., Hon C.M., Madheswaran T., Gupta G., Pan-neerselvam J., Lakshmi T., Singh S.K., Gulati M. (2021). Anti-inflammatory and anticancer activities of Naringenin-loaded liquid crystalline nanoparticles in vitro. J. Food Biochem..

[42] Martínez-Rodríguez O.P., González-Torres A., Álvarez-Salas L.M., Hernández-Sánchez H., García-Pérez B.E., Thompson-Bonilla M.R., Jaramillo-Flores M.E. (2020). Effect of naringenin and its combination with cisplatin in cell death, proliferation and invasion of cervical cancer sphe-roids. RSC Adv..

[43] Liao A.C.H., Kuo C.C., Huang Y.C., Yeh C.W., Hseu Y.C., Liu J.Y., Hsu L.S. (2014). Naringenin inhibits migration of bladder cancer cells through downregulation of AKT and MMP-2. Mol. Med. Rep..

[44] Han K.Y., Chen P.-N., Hong M.-C., Hseu Y.-C., Chen K.-M., Hsu L.-S., Chen W.-J. (2018). Naringenin attenuated prostate Cancer invasion via reversal of epithelial–to–Mesenchymal tran-sition and inhibited uPA activity. Anticancer Res..

[45] Bao L., Liu F., Guo H.B., Li Y., Tan B.B., Zhang W.X., Peng Y.H. (2016). Naringenin inhibits proliferation, migration, and invasion as well as induces apoptosis of gastric cancer SGC7901 cell line by downregulation of AKT pathway. Tumor Biol..

[46] Park H.J., Choi Y.J., Lee J.H., Nam M.J. (2017). Naringenin causes ASK1-induced apoptosis via reactive oxygen species in human pancreatic cancer cells. Food Chem. Toxicol..

[47] Ahamad M.S., Siddiqui S., Jafri A., Ahmad S., Afzal M., Arshad M. (2014). Induction of apopto-sis and antiproliferative activity of naringenin in human epidermoid carcinoma cell through ROS generation and cell cycle arrest. PLoS ONE.

[48] Zeng W., Jin L., Zhang F., Zhang C., Liang W. (2018). Naringenin as a potential immunomodu-lator in therapeutics. Pharmacol. Res..

[49] Ke J., Banh T., Hsiao Y., Cole R., Straka S., Yee L., Belury M. (2017). Citrus flavonoid naringenin reduces mammary tumor cell viability, adipose mass, and adipose inflammation in obese ovariectomized mice. Mol. Nutr. Food Res..

[50] Qin L., Jin L., Lu L., Lu X., Zhang C., Zhang F., Liang W. (2011). Naringenin reduces lung me-tastasis in a breast cancer resection model. Protein Cell.

[51] Gontero P., Marra G., Soria F., Oderda M., Zitella A., Baratta F., Chiorino G., Gregnanin I., Daniele L., Cattel L. (2015). A randomized double-blind placebo controlled phase I–II study on clinical and molecular effects of dietary supplements in men with precancerous prostatic lesions. Chemoprevention or “chemopromotion”?. Prostate.

[52] Lippman S.M., Klein E.A., Goodman P.J., Lucia M.S., Thompson I.M., Ford L.G., Parnes H.L., Minasian L.M., Gaziano J.M., Hartline J.A. (2009). Effect of Selenium and Vitamin E on Risk of Prostate Cancer and Other Cancers: The Selenium and Vitamin E Cancer Prevention Trial (SELECT). JAMA.

[53] Yildiz A., Kaya Y., Tanriverdi O. (2019). Effect of the Interaction Between Selenium and Zinc on DNA Repair in Association with Cancer Prevention. J. Cancer Prev..

[54] Quesada I.M., Bustos M., Blay M., Pujadas G., Ardevol A., Salvado M.J., Blade C., Arola L., Fernandez-Larrea J. (2011). Dietary catechins and procyanidins modulate zinc homeostasis in hu-man HepG2 cells. J. Nutr. Biochem..

[55] Malhotra A., Nair P., Dhawan D.K. (2011). Curcumin and resveratrol synergistically stimulate p21 and regulate cox-2 by maintaining adequate zinc levels during lung carcinogenesis. Eur. J. Cancer Prev..

[56] Schram K.H. (1998). Urinary nucleosides. Mass Spectrom. Rev..

[57] Raina M., Ibba M. (2014). tRNAs as regulators of biological processes. Front. Genet..

[58] Dunn D.B., Smith J.D. (1955). Occurrence of a New Base in the Deoxyribonucleic Acid of a Strain of Bacterium Coli. Nature.

[59] Dunn D.B., Smith J.D. (1958). The occurrence of 6-methylaminopurine in deoxyribonucleic acids. Biochem. J..

[60] Li X., Zhang Z., Luo X., Schrier J., Yang A.D., Wu T.P. (2021). The exploration of N6-deoxy-adenosine methylation in mammalian genomes. Protein Cell.

[61] Wu B., Luo L., Gao X.J. (2016). Cas9-triggered chain ablation of cas9 as a gene drive brake. Nat. Biotechnol..

[62] Yao C.K., Liu Y.T., Lee I.C., Wang Y.T., Wu P.Y. (2017). A Ca^2+^ channel differentially regulates Clathrin-mediated and activity-dependent bulk endocytosis. PLoS Biol..

[63] Xie T., Ho M.C.W., Liu Q., Horiuchi W., Lin C.C., Task D., Luan H., White B.H., Pot-ter C.J., Wu M.N. (2018). A Genetic Toolkit for Dissecting Dopamine Circuit Function in Drosophila. Cell Rep..

[64] Li F., Yi Y., Miao Y., Long W., Long T., Chen S., Cheng W., Zou C., Zheng Y., Wu X. (2019). N6-Methyladenosine Modulates Nonsense-Mediated mRNA Decay in Human Glioblastoma. Cancer Res..

[65] Shen H., Lan Y., Zhao Y., Shi Y., Jin J., Xie W. (2020). The emerging roles of N6-methyladeno-sine RNA methylation in human cancers. Biomark. Res..

[66] Zhang Y., Liu S., Zhao T., Dang C. (2021). METTL3-mediated m6A modification of Bcl-2 mRNA promotes non-small cell lung cancer progression. Oncol. Rep..

[67] Wang H., Xu B., Shi J. (2020). N6-methyladenosine METTL3 promotes the breast cancer progression via targeting Bcl-2. Gene.

[68] Liu J., Harada B.T., He C. (2019). Regulation of gene expression by N6-methyladenosine in cancer. Trends Cell Biol..

[69] Xiao H., Fan X., Zhang R., Wu G. (2021). Upregulated N6-Methyladenosine RNA in Peripheral Blood: Potential Diagnostic Biomarker for Breast Cancer. Cancer Res. Treat.

[70] Heckmann B.L., Yang X., Zhang X., Liu J. (2013). The autophagic inhibitor 3-methyladenine po-tently stimulates PKA-dependent lipolysis in adipocytes. Br. J. Pharmacol..

[71] Yoon J.H., Choudhury J.R., Park J., Prakash S., Prakash L. (2017). Translesion synthesis DNA polymerases pro-mote error-free replication through the minor-groove DNA adduct 3-deaza-3-methyladenine. J. Biol. Chem..

[72] Boysen G., Pachkowski B.F., Nakamura J., Swenberg J.A. (2009). The formation and biological significance of N7-guanine adducts. Mutat. Res..

[73] Rinne M.L., He Y., Pachkowski B.F., Nakamura J., Kelley M.R. (2005). N-methylpurine DNA glycosylase overex-pression increases alkylation sensitivity by rapidly removing non-toxic 7-methylguanine adducts. Nucleic Acids Res..

[74] Hou H., Zhang Y., Huang Y., Qiyi Y., Lv L., Zhang T., Chen D., Hao Q., Shi Q. (2012). Inhibi-tors of phosphatidylinositol 3′-kinases promote mitotic cell death in HeLa cells. PLoS ONE.

